# A Multi‑Center, Open‑Label, Single‑Arm Trial to Evaluate the Efficacy, Pharmacokinetics, and Safety and Tolerability of IGSC 20% in Subjects with Primary Immunodeficiency

**DOI:** 10.1007/s10875-021-01181-6

**Published:** 2022-01-01

**Authors:** Manuel Santamaria, Olaf Neth, Jo A. Douglass, Gergely Krivan, Robin Kobbe, Ewa Bernatowska, Sofia Grigoriadou, Claire Bethune, Anita Chandra, Gerd Horneff, Michael Borte, Anja Sonnenschein, Pavlina Kralickova, Silvia Sánchez Ramón, Daman Langguth, Luis Ignacio Gonzalez-Granado, Laia Alsina, Montse Querolt, Rhonda Griffin, Carrie Hames, Elsa Mondou, Jeffrey Price, Ana Sanz, Jiang Lin

**Affiliations:** 1 Unidad de Inmunologia Clinica, Hospital Universitario Reina Sofía, Facultad de Medicina, Universidad de Cordoba, Cordoba, Spain; 2 Pediatric Infectious Diseases, Rheumatology and Immunology Unit, Hospital Universitario Virgen del Rocío, Instituto de Biomedicina de Sevilla, Universidad de Sevilla/CSIC, Red de Investigación Traslacional en Infectología Pediátrica RITIP, IBiSSeville, Spain; 3 Department of Immunology and Allergy, The Royal Melbourne Hospital, Melbourne, VIC Australia; 4 Department of Medicine, The University of Melbourne, Melbourne, VIC Australia; 5 Department for Paediatric Haematology and Hemopoietic Stem Cell Transplantation, Central Hospital of Southern Pest – National Institute of Hematology and Infectious Diseases, Budapest, Hungary; 6 First Department of Medicine, Division of Infectious Diseases, University Medical Center Hamburg-Eppendorf, Hamburg, Germany; 7 Department of Immunology, Children’s Memorial Health Institute, Warsaw, Poland; 8 Department of Immunology, The Royal London Hospital, Barts Health NHS Trust, London, UK; 9 Peninsula Immunology and Allergy Service, University Hospitals Plymouth, Plymouth, UK; 10 Department of Medicine, University of Cambridge, Cambridge, UK; 11 Asklepios Kinderklinik Sankt Augustin, Sankt Augustin, Germany; 12 University Hospital of Cologne, Cologne, Germany; 13 Klinikum St Georg GmbH, Klinik für Kinder‐ und Jugendmedizin, Leipzig, Germany; 14 Department of Pediatric Immunology and Rheumatology, University Medical Center of Johannes Gutenberg University Mainz, Mainz, Germany; 15 Department of Allergology and Clinical Immunology, Faculty of Medicine, Charles University and University Hospital in Hradec Kralove, Hradec Kralove, Czechia; 16 Servicio de Inmunología, Hospital Clínico San Carlos, Madrid, Spain; 17 Immunology Department, Sullivan Nicolaides Pathology, Brisbane, Australia; 18 Primary Immunodeficiencies Unit, Hospital Universitario 12 de Octubre and Department of Public and Maternal - Child Health, Faculty of Medicine, Complutense University of Madrid, Madrid, Spain; 19 Clinical Immunology and Primary Immunodeficiencies Unit, Pediatric Allergy and Clinical Immunology Department, Hospital Sant Joan de Déu, Barcelona, Spain; 20 Institut de Recerca Sant Joan de Déu, Barcelona, Spain; 21 Universitat de Barcelona, Barcelona, Spain; 22 Grifols Bioscience Research Group, Sant Cugat del Vallès, Barcelona, Spain; 23 Grifols Bioscience Research Group, Research Triangle Park, NC USA

**Keywords:** Primary immunodeficiency, immunoglobulin replacement therapy, subcutaneous, 20% immunoglobulin, GTI1503

## Abstract

**Purpose:**

The purpose of this phase 3 study was to evaluate the efficacy, pharmacokinetics (PK), and safety of Immune Globulin Subcutaneous (Human), 20% Caprylate/Chromatography Purified (IGSC 20%) in patients with primary immunodeficiency (PI).

**Methods:**

Immunoglobulin treatment-experienced subjects with PI received 52 weeks of IGSC 20% given weekly at the same dose as the subject’s previous IgG regimen (DAF 1:1); the minimum dose was 100 mg/kg/week. The primary endpoint was serious bacterial infections (SBIs [null vs alternative hypothesis: SBI rate per person per year ≥ 1 vs < 1]). IgG subclasses and specific pathogen antibody levels were also measured.

**Results:**

Sixty-one subjects (19 children [≤ 12 years], 10 adolescents [> 12–16 years], and 32 adults) were enrolled. The rate of SBIs per person per year was 0.017. The 1-sided 99% upper confidence limit was 0.036 (< 1), and the null hypothesis was rejected. The rate of hospitalization due to infection per person per year was 0.017 (2-sided 95% confidence interval: 0.008–0.033) overall. The mean trough total IgG concentrations were comparable to the previous IgG replacement regimen. The average of the individual mean trough ratios (IGSC 20%:previous regimen) was 1.078 (range: 0.83–1.54). The average steady-state mean trough IgG concentrations were 947.64 and 891.37 mg/dL, respectively. Seven subjects had serious treatment-emergent adverse events (TEAEs); none was drug-related. The rate of all TEAEs, including local infusion site reactions, during 3045 IGSC 20% infusions was 0.135. Most TEAEs were mild or moderate.

**Conclusions:**

IGSC 20% demonstrated efficacy and good safety and tolerability in subjects with PI.

**Supplementary Information:**

The online version contains supplementary material available at 10.1007/s10875-021-01181-6.

## Introduction

Immunoglobulin G (IgG) replacement therapy is widely used in the treatment of patients with infections associated with primary immunodeficiency (PI) [1–4]. While intravenous (IV) infusion of immune globulin (IVIG) is a well-established approach, some patients (e.g., poor venous access, at risk of systemic adverse events [AEs]) may benefit from a subcutaneous (SC) route of administration [5, 6]. Hence, products for SC delivery of immune globulin (SCIG) are effective in the prevention of infections in adults, adolescents, and children (≤ 12 years). SCIG also offers the possibility of administration in a home setting [5, 7–16]. The advantages of SCIG relative to IVIG—convenience, ease of adoption and use, and greater independence and autonomy [3, 4, 11, 16]—have increased access to safe and effective IgG treatments for many patients with PI [17–24].

A SCIG product, Immune Globulin Subcutaneous (Human), 20% Caprylate/Chromatography Purified (IGSC 20% [Grifols Bioscience Research Group, Research Triangle Park, NC, USA]) for IgG replacement treatment for patients with PI has been developed. In previous research, IGSC-C 20% was shown to be bioequivalent to IGIV-C 10% and was well tolerated, with a safety profile comparable with IGIV-C 10% [25]. The purpose of this study was to evaluate the efficacy, pharmacokinetics (PK), and safety of IGSC 20% in patients with PI. Its primary objective, developed in accordance with the European Medicines Agency (EMA) guideline (July 2015) [26], was to evaluate whether weekly administration of IGSC 20% over 1 year resulted in less than 1 serious bacterial infection (SBI) per subject per year.

## Methods

### Study Design

This phase 3 study (GTI1503; EudraCT #: 2015–003,290-15; clinicaltrials.gov identifier NCT02806986) was a multicenter, single-arm, open-label study conducted in 8 countries at 22 participating centers in the United Kingdom, Hungary, Germany, Czech Republic, Spain, France, Poland, and Australia. Screened and/or enrolled subjects had PI with impaired antibody production, including combined immunodeficiency syndromes (e.g., Hyper-IgM syndrome).

The study involved 2 phases: a screening/previous regimen phase and an IGSC 20% treatment phase. During the screening/previous regimen phase, subjects continued to take their existing IVIG or SCIG treatment (previous regimen) and underwent measurement of 2 IgG trough levels. If all eligibility criteria were met, subjects completed the screening/previous regimen phase and entered the treatment phase. In the treatment phase, subjects self-administered IGSC 20% at home (caregivers administered IGSC 20% to young children). They had been trained and observed at 3 successive clinic visits to assure adequate understanding and technical expertise. Following baseline assessments, subjects entering the study on IVIG commenced treatment with SC IGSC 20% using a 1:1 dose adjustment factor (DAF). They continued to receive SC IGSC 20% as a weekly dose for 52 weeks and completed a final follow-up visit at week 53.

The study protocol was approved by Institutional Review Boards/Ethics Committees/Research Ethics Boards at all participating institutions and by regulatory authorities in all countries in which it was conducted. All subjects provided written informed consent before any study-related procedures were undertaken, and the clinical study was conducted in full conformance with applicable local laws and regulations, the Declaration of Helsinki, and the international standards of Good Clinical Practice.

### Study Population

Eligible subjects included children, adolescent, and adult males and females between the ages of 2 and 75 years (inclusive) at screening, with a documented and confirmed pre-existing diagnosis of PI with features of hypogammaglobulinemia requiring IgG replacement therapy, including but not limited to humoral-based immunodeficiency syndromes and combined immunodeficiency syndromes without lymphocytopenia [27]. Subjects could not have had an SBI within the last 3 months prior to screening and up to the time of the baseline visit, as well as during the screening/previous regimen phase and prior to the first study treatment. Subjects had to be on a stable regimen (dose and dosage) of IVIG or SCIG for at least 3 consecutive months. Those receiving IVIG prior to study participation must have received a dosage of at least 200 mg/kg per infusion. Subjects also had to have documentation (within the previous 6 months) of an IgG trough level of ≥ 500 mg/dL on the current IgG replacement therapy regimen, and they had to have screening/pre-baseline trough IgG levels of at least 500 mg/dL.

Subjects were ineligible to participate in the study if they had clinical evidence of any significant acute or chronic disease that may have interfered with successful completion of the trial or placed the subject at undue medical risk. They were also excluded if they had a known serious adverse reaction to immunoglobulin or any severe anaphylactic reaction to blood or any blood-derived product; a history of blistering skin disease, clinically significant thrombocytopenia, bleeding disorder, diffuse rash, recurrent skin infections, or other disorders where SC therapy would be contraindicated; isolated IgG subclass deficiency, isolated specific antibody deficiency disorder, or transient hypogammaglobulinemia of infancy; or a known selective IgA deficiency with or without antibodies to IgA. Females of childbearing potential who were pregnant, had a positive pregnancy test at screening (serum) or baseline (urine) (human chorionic gonadotropin-based assay), were breastfeeding, or unwilling to practice a highly effective method of contraception throughout the study were excluded. Subjects with significant proteinuria (dipstick proteinuria ≥ 3 + , known urinary protein loss > 1 g/24 h, or nephrotic syndrome), a history of acute renal failure, severe renal impairment (blood urea nitrogen or creatinine more than 2.5 times the upper limit of normal), and/or on dialysis were ineligible, as were subjects whose aspartate aminotransferase or alanine aminotransferase levels exceeded 2.5 times the upper limit of normal and those with hemoglobin < 9 g/dL at screening. Subjects were also excluded if they had a history of (1 year before screening or 2 lifetime episodes) or current hepatitis B virus; hepatitis C virus; deep venous thrombosis; or thromboembolism; were receiving anti-coagulation therapy that would make SC administration inadvisable; had a known hyperviscosity syndrome; had an acquired medical condition that was known to cause secondary immune deficiency; were positive for human immunodeficiency virus; had uncontrolled arterial hypertension; were receiving immunosuppressants, immunomodulators, or long-term systemic corticosteroids (i.e., daily dose > 1 mg of prednisone equivalent/kg/day for > 30 days); were known to abuse psychoactive substances or prescription drugs. Subjects who had participated in another clinical trial within 30 days prior to screening or received any investigational blood product (except other IgG products) within the previous 3 months, as well as those who were unwilling to comply with any aspect of the protocol for the duration of the study, were considered ineligible.

### Treatments

The study treatment was Grifols IGSC 20%, a sterile liquid formulation containing 20% human immune globulin formulated in 0.16–0.26 M glycine and 10–40 parts per million polysorbate 80 at pH 4.1–4.8. IGSC 20% is manufactured using the same process as for Gamunex 10%, except that ultrafiltration is added to achieve the 20% IgG concentration.

Eligible subjects, including those on IVIG, received IGSC 20% at the same weekly equivalent dose as their previous IgG replacement regimen. Beginning 1 week after the last IVIG dose or at the time when their next commercial SCIG dose was due, weekly IGSC 20% was administered for 52 weeks at a minimum dose of IGSC 20% of 100 mg/kg/week.

### Assessments

The primary efficacy endpoint was the number and frequency of SBIs, as defined by the EMA guideline and FDA guidance for analyzing annualized SBI rate in primary efficacy analysis [26, 28]. Secondary efficacy endpoints included the rate of infection of any kind (serious and non-serious); antibiotic treatment (oral, parenteral, oral plus parenteral, prophylactic, and therapeutic); hospitalizations due to infection; days lost from work/school/daily activities due to infections and related treatment; and mean total IgG trough levels during steady state. Additional parameters included average trough concentration of IgG subclasses (IgG1, IgG2, IgG3, and IgG4) and concentration of antibody levels to *S. pneumoniae*, *H. influenzae*, *C. tetani* (tetanus), and measles.

The study included a subset of adults (planned n ~ 20) for PK profiling, with serial samples collected immediately before and after SC#17 infusion at steady state over a 7-day period. Parameters included area under the concentration versus time curve from time 0 to 7 days (*AUC*_0-7 days_), the observed maximum total IgG concentration (*C*_max_), and the observed time to reach the first observed maximum total IgG concentration (*t*_max_) and were determined by standard non-compartmental methods using Phoenix WinNonlin (Certara USA, Inc., Princeton, NJ, USA).

Safety was evaluated monthly for clinical findings (AE and infusion site reaction assessments, physical examination, and vital signs) and laboratory testing. To confirm the appropriate use of study treatment throughout the study, the self-administration of IGSC 20% was observed at scheduled clinic visits.

### Statistical Methods

The efficacy-evaluable and safety populations included subjects who received any amount of IGSC 20%. The IgG population included subjects who received IGSC 20% and had sufficient trough IgG concentration data to allow the calculation of steady-state mean trough IgG in the IGSC 20% phase. The PK population included subjects in the adult PK subset who received IGSC 20% and had sufficient serial IgG concentration vs. time data to allow the calculation of PK parameters.

Descriptive statistics were calculated as appropriate for the type of endpoints (continuous/quantitative or categorical/qualitative). Geometric mean and 90% confidence interval (CI) were additionally calculated for PK parameters (except *t*_max_). For infection-related efficacy endpoints, the rate per person per year was calculated as the total number of events or days divided by the total duration of exposure (in years) over all subjects.

The primary efficacy analysis tested the null hypothesis that the SBI rate per person per year during IGSC 20% treatment was greater than or equal to 1 (the recommended standard rate) vs. the alternative hypothesis that the rate was less than 1, at a 1-sided alpha level of 0.01. The generalized linear model procedure for Poisson regression with log link was used to estimate the SBI rate per person per year for IGSC 20% and its one-sided 99% upper confidence limit (CL) (or equivalently, the upper limit of the two-sided 98% CI). The natural log-transformed person-year was used in the generalized linear model as an offset variable with only the intercept term included in the model. The estimated intercept term and the upper limit of its 2-sided 98% CI were back-transformed by using the natural exponential function, to provide the point estimate of the SBI rate per person per year for IGSC 20% and its one-sided 99% upper CL. If the 1-sided 99% upper CL was less than 1, then the null hypothesis was rejected at one-sided *α* = 0.01 level.

Secondary and other infection-related efficacy endpoints were analyzed with the same methods as in the primary efficacy analysis. For these endpoints, the 2-sided 95% CIs (equivalent to a 2-sided alpha level of 0.05) were derived from the generalized linear model procedure for Poisson regression.

Mean trough total IgG concentration for the IGSC 20% treatment phase was calculated as the average of all steady-state trough concentrations measured during the IGSC 20% treatment phase at the following visits: week 17, 18, 20, 24, 28, 32, 36, 40, 44, 48, 52, and 53. Comparison was made to the mean trough total IgG concentration for the previous IgG regimen (either IVIG or other SCIG products), calculated as the average of 2 trough concentrations collected during the previous regimen phase.

Sample size was determined based on the primary endpoint. Assuming that the true rate of the SBIs was 0.25 per person per year, 40 subjects treated for 1 year with IGSC 20% would provide at least 90% power to reject the null hypothesis of an SBI rate greater than or equal to 1.0 per person per year, using a 1-sided test at the 0.01 level. In order to assure a total of 40 PI subjects, including 20 adult and 20 pediatric evaluable subjects, enrollment of approximately 60 subjects was planned.

## Results

### Subjects

Sixty-one subjects were recruited and participated in GTI1503 from 29 June 2016 through 15 May 2019 at 15 study centers in North America and Europe. All 61 subjects received IGSC 20% and were included in the safety and efficacy evaluable populations. Data from 59 subjects were valid for steady-state trough IgG concentration analysis (IgG population). A total of 29 adults participated in the PK subset, and 27 were valid for the PK analysis (PK population).

**Fig. 1:**
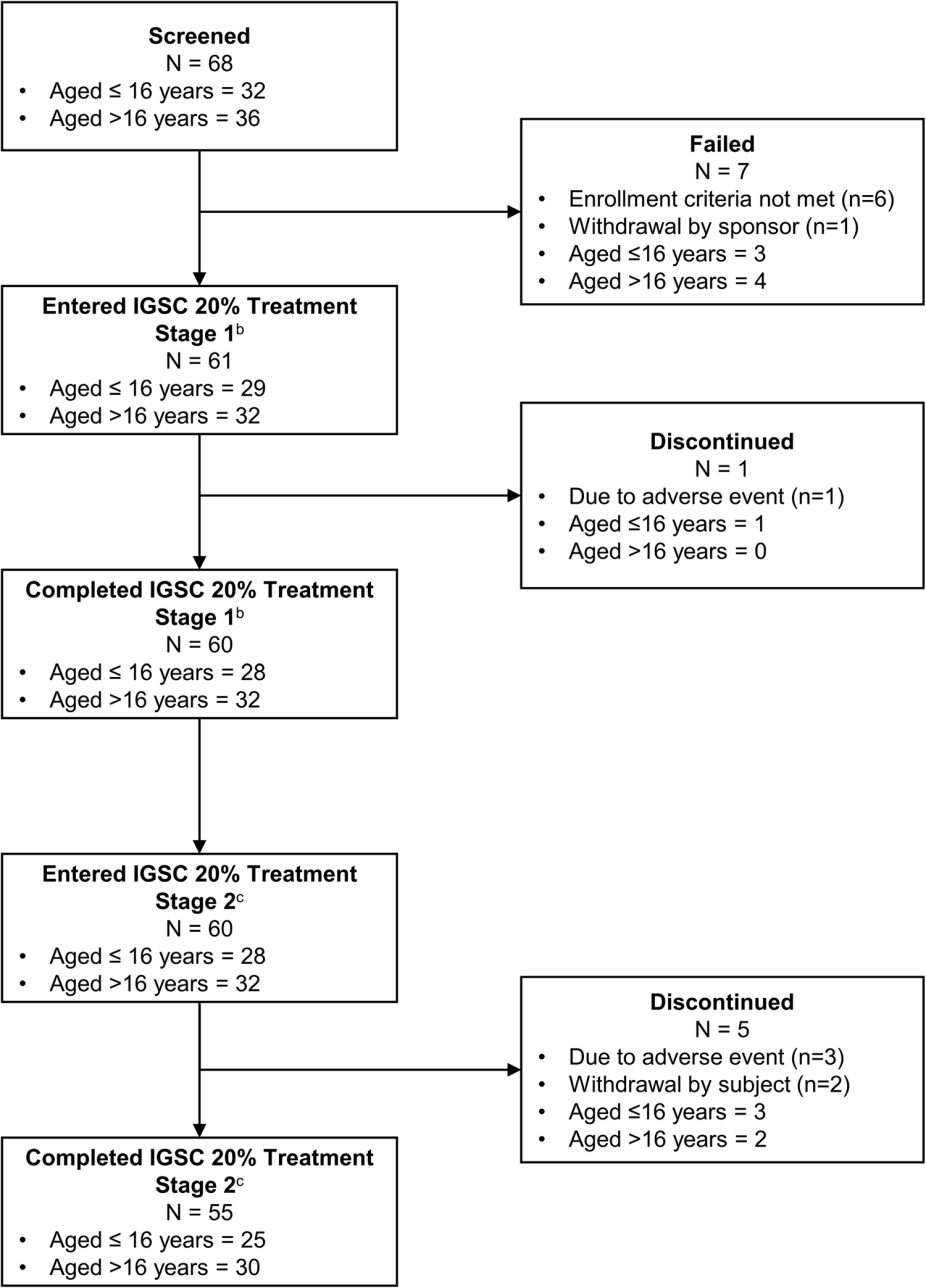
Subject Disposition^a^. ^a^One subject who was screened and entered into the previous regimen phase was withdrawn at sponsor’s request prior to receiving any amount of IGSC 20% due to reported serious adverse events (non-productive cough with accompanying cyanosis, hypoxemia, interstitial pneumonitis) ^b^Refers to the first 13 weeks of treatment ^c^Refers to the next 39 weeks of treatment

The disposition of subjects is depicted in Fig. 1. Sixty-eight subjects were screened, and 61 met eligibility criteria and were suitable for participation. Of these, 55 (90.2%) completed the study and 6 (9.8%) discontinued, 4 due to treatment-emergent adverse events (TEAEs), and 2 due to subjects’ own decision.

The study population was 52.5% (32/61) adults and 47.5% (29/61) children and adolescents; the 29 non-adults were aged 2–5 years (17.2% [5/29]), > 5 to 12 years (48.3% [14/29]), and > 12 to 16 years (34.5% [10/29]). The majority of subjects were white (93.4% [57/61]), and most were male (68.9% [42/61]). The most common types of PI were common variable immunodeficiency (CVID, 63.9% [39/61]) and X-linked agammaglobulinemia (21.3% [13/61]). Median (range) time since PI diagnosis was 5.99 (0.3–38.9) years. The previous regimen for most subjects (65.6% [40/61]) was IVIG; 34.4% (21/61) of subjects were receiving SCIG at entry. Those whose previous regimen was IVIG were more likely to be receiving treatment every 4 weeks (37.7% [23/61]) than every 3 weeks (27.9% [17/61]). Demographic and other baseline characteristics, as well as subjects’ treatment during the screening/previous regimen phase, are summarized in Table 1.

**Table.1:** Demographics, disease characteristics, and entry IgG regimen in the efficacy-evaluable population (*N* = 61)

Sex, *n* (%)	
Female	19 (31.1)
Male	42 (68.9)
Age, years	
Mean (SD)	27.3 (19.97)
Median (range)	17.0 (2, 69)
Weight, kg	
Mean (SD)	57.59 (24.27)
Median (range)	57.20 (13.9, 133.0)
Race, *n* (%)	
White	57 (93.4)
American Indian or Alaska Native	2 (3.3)
Unknown	2 (3.3)
Time since primary immunodeficiency diagnosis, years	
Mean (SD)	8.99 (7.899)
Median (range)	5.99 (0.3, 38.9)
Primary immunodeficiency, *n* (%)	
Common variable immunodeficiency	39 (63.9)
X-linked agammaglobulinemia	13 (21.3)
Primary hypogammaglobulinemia	8 (13.1)
Hyper IgM immunodeficiency syndrome	1 (1.6)
Entry IgG replacement regimen, *n* (%)	
Entered on IVIG	40 (65.6)
Entered on SCIG	21 (34.4)
Weekly equivalent dose of previous regimen at study entry, mg/kg	
Entered on IVIG (*n* = 40)	
Mean (SD)	127.41 (36.298)
Median (range)	123.75 (57.8, 209.0)
Entered on SCIG (*n* = 21)	
Mean (SD)	110.10 (33.465)
Median (range)	105.00 (66.0, 169.0)
Total (*n* = 61)	
Mean (SD)	121.45 (36.034)
Median (range)	116.67 (57.8, 209.0)

*IVIG*, intravenous immunoglobulin; *SCIG*, subcutaneous immunoglobulin; *SD*, standard deviation

### Compliance and SC Administration Characteristics

In total, 3045 IGSC 20% infusions were administered, and mean (SD) infusion compliance was 99.9 (0.59)%. The median (interquartile range [IQR]) duration of each weekly infusion was 1 (0.77–1.53) hour, the median (IQR) infusion rate was 21 (13.73–26.27) mL/h/site, and a median of 2 sites was used per infusion. Based on the IGSC 20% infusions with analyzable data (*N* = 3044), 261 infusions were administered to 14 subjects at infusion rates > 25 mL/h/site; the median (IQR) and mean (SD) infusion rates in this subgroup were 37.5 (33.83–44.97) and 39.4 (8.25) mL/h/site, respectively. Mean (SD) IGSC 20% dose per weekly infusion was 125.5 (28.46) mg/kg/week. Infusion parameters are summarized in Table 2.

**Table.2:** IGSC 20% dose and infusion characteristics in the safety population^a^

	Median (interquartile range)	Mean (SD)
Weekly dose per infusion, mg/kg	113.0 (106.3–144.7)	125.5 (28.46)
Volume per infusion, mL	35.5 (25.4–48.6)	37.0 (17.20)
Duration per infusion, hours	1.00 (0.77–1.53)	1.15 (0.57)
Infusion sites per infusion,^b^ n	2.0	2.0 (0.75)
Volume infused per site per infusion, mL/site	19.0 (14.14–24.46)	19.3 (7.64)
Infusion rate per site per infusion, mL/h/site	21.0 (13.73–26.27)	20.0 (9.29)
Dose conversion factor^c^	1.000 (0.96–1.17)	1.065 (0.162)

*SD*, standard deviation

^a^3044 infusions in 61 subjects

^b^Since the number of infusion sites must be an integer, the interquartile range was not calculated

^c^IGSC 20% weekly dose/weekly dose equivalent of previous IgG regimen

### Efficacy

For the primary efficacy endpoint of SBIs, only 1 subject (1.6% [1/61]), a child, experienced an infection that met the criteria for an SBI. The event of pneumonia was successfully treated on an outpatient basis with oral antibiotics, and the subject recovered within 4 days.

The total duration of IGSC 20% exposure was 58.40 subject-years. The rate of SBI was 0.017 (2-sided 98% CI: 0.006–0.036) per person per year overall. Of note, since the upper limit of the 2-sided 98% CI was statistically equivalent to the 1-sided 99% upper CL (0.036) and less than 1, the null hypothesis was rejected at a 1-sided *α* = 0.01 level, and the primary efficacy endpoint was met. A summary of infections and associated parameters (primary and secondary endpoint variables) is provided in Table 3.

**Table.3:** Primary and secondary endpoints, summary of infections, and associated events in the efficacy-evaluable population

Subjects, *n*	61
Subject-years on treatment	58.40
Annual SBIs per subject-year^a^ (98% CI)	0.017 (0.006–0.036)
Annual infections per subject-year (95% CI)	2.397 (1.824–3.079)
Annual validated infections per subject-year^b^ (95% CI)	0.616 (0.401–0.898)
Days on antibiotics (prophylactic) per subject-year (95% CI)	44.432 (26.351–69.339)
Days on antibiotics (therapeutic) per subject-year (95% CI)	8.904 (5.949–12.705)
Days missed from work/school/unable to perform normal daily activities due to infections per subject-year (95% CI)	4.983 (3.064–7.572)
Hospitalizations due to infections per subject-year^c^ (95% CI)	0.017 (0.008–0.033)

*CI*, confidence interval; *SBI*, serious bacterial infection

^a^1 outpatient pneumonia

^b^Validated infections, as documented by positive radiograph, fever (> 38 °C oral or > 39 °C rectal), culture, or diagnostic testing (e.g., rapid streptococcal antigen detection test) for microorganisms (e.g., bacterial, viral, fungal, or protozoal pathogens)

^c^1 inpatient urinary tract infection

Rate of events/days per subject-year is calculated as the total number of events/days divided by the total duration of exposure in years across all subjects

2-sided 98% and 95% confidence interval (CI) is determined from a generalized linear model for Poisson regression for the log-transformed number of events/days with log-transformed duration of exposure in years as an offset variable

During the IGSC 20% treatment phase, the rate of hospitalization due to infection was low (1.6% [1/61]), with only 1 event of urinary tract infection. The rate of hospitalization due to infection was 0.017 (2-sided 95% CI: 0.008–0.033) per person per year overall. The rate of infections of any kind (all severities) was 2.397 (2-sided 95% CI: 1.824–3.079) per person per year overall. Thirty-six validated infections were documented by positive radiograph, fever, culture, or positive diagnostic test for microorganisms during the treatment phase, and the rate of validated infections per person per year was 0.616 (2-sided 95% CI: 0.401–0.898).

Antibiotic usage was categorized as prophylactic or therapeutic. During the IGSC 20% treatment phase, the number of days on prophylactic antibiotics per person per year was 44.432 (2-sided 95% CI: 26.351–69.339). Overall, the number of days on therapeutic antibiotics per person per year was 8.904 (2-sided 95% CI: 5.949–12.705).

The number of days of work/school/daily activities missed per subject year due to infections and related treatment was 4.983 (2-sided 95% CI: 3.064–7.572) overall. Results for disability parameters with regard to infections and associated events were consistent across all age categories.

In the IgG population (*n* = 59), as shown in Table 4, the average of the steady-state mean trough concentrations of total IgG for all subjects during the previous regimen phase was 891.37 mg/dL; the IGSC 20% treatment phase results were generally comparable (947.64 mg/dL). The average mean trough ratio (treatment:previous regimen) was 1.078. A summary of the derived PK parameters (*AUC*, *C*_max_, and *t*_max_) in the PK population (*n* = 27) is additionally presented in Table 4.

**Table.4:** Mean trough IgG levels during previous regimen and on IGSC 20% treatment and pharmacokinetic parameters in adult subjects

Mean trough (mg/dL) in IgG population^a^			
	IgG mean trough		Mean trough ratio
	Previous regimen^b^	IGSC 20% SC^c^	IGSC 20% SC/ Previous regimen
*n*	59	59	59
Mean (SD)	891.37 (165.943)	947.64 (150.262)	1.078 (0.1425)
CV%	18.6	15.9	13.22
Median (range)	874.00 (516.5, 1255.0)	909.10 (629.2, 1340.8)	1.050 (0.83, 1.54)
Geometric mean	875.96	936.48	1.069
PK parameters in the adult subset^d^			
	*AUC*_0-7 days_ (h*mg/dL)	*C*_max_ (mg/dL)	*T*_max_ (hours)
*n*	27	27	27
Mean (SD)	177,445.7 (31,081.89)	1126.6 (190.11)	50.78 (44.596)
CV%	18	17	87.8
Median	172,369.0	1080.0	68.80
Geometric mean	175,002.1	1112.2	—
90% CI for geometric mean	165,652.5–184,879.5	1055.1–1172.4	—

*PK*, pharmacokinetic; *AUC*, area under the concentration–time curve; *C*_*max*_, maximum concentration; *CV*, coefficient of variation; *SD*, standard deviation; *T*_*max*_, time to first observed C_max_

^a^59 subjects were valid for steady-state IgG trough analysis; 2 subjects were excluded because they withdrew from study before week 17 and did not have any steady-state trough IgG samples to include in the calculation

^b^Average of the 2 trough concentrations during the previous regimen phase

^c^Average of trough concentrations at the following weeks during PK steady state: 17, 18, 20, 24, 28, 32, 36, 40, 44, 48, 52, and 53

^d^27 subjects from the PK population with sufficient serial PK concentration data

The trough levels of IgG subclasses IgG1, IgG2, IgG3, and IgG4 reached a steady state in the IGSC 20% treatment phase by at least week 13, and the overall levels of IgG subclasses were consistent with those observed during the previous regimen phase. For example, the average IgG1 trough level was 639.0 mg/dL at week 13 of the IGSC 20% treatment phase and 561.5 mg/dL during the previous regimen phase. No preferential absorption or loss of IgG subclasses was observed, and the trough concentrations of IgG subclasses were generally consistent across the different age groups.

During IGSC 20% treatment, the mean trough antibody levels were above protective thresholds for *H. Influenzae* (protective ≥ 1.0 µg/mL; 0.15 µg/mL protective for invasive disease [29]) and anti-*tetanus* toxoid (> 0.1 IU/mL [30]). Measles mean titer values and all individual subject titers were above protective trough titers for PI patients (threshold: 0.24 IU/mL [31]). Of the 23 *S. pneumoniae* serotypes evaluated for antibody levels, mean antibody concentrations were generally above the protective levels for noninvasive disease (≥ 1.3 µg/mL) for most serotypes, and they exceeded the protective threshold for invasive disease (≥ 0.2 µg/mL) for all serotypes [32–34]. Trough IgG concentrations against all pathogens analyzed were generally consistent across the different age groups.

### Safety

As presented in Table 5, the TEAEs resulting in the discontinuation of 4 subjects included anxiety (not related to IGSC 20%) in a 2-year-old child, aortic valve incompetence related to congenital heart disease (not related to IGSC 20%), nephrotic syndrome (which evolved during the study and was unrelated to IGSC 20%), and subcutaneous fibroma. The subject with nephrotic syndrome was discontinued upon diagnosis. Seven subjects had 7 treatment-emergent serious AEs (SAEs), none of which were considered to be related to treatment with IGSC 20%: urinary tract infection, nephrotic syndrome, luxating patella, thrombocytopenia (subject history of immune thrombocytopenia), aortic valve incompetence requiring cardiac surgery, medical device site joint pain (orthopedic arthrodesis screw), and 1 case of outpatient pneumonia (met criteria for the primary efficacy endpoint [SBI]). The subject with the SAE of thrombocytopenia withdrew of their own volition. Local infusion site reactions reported as TEAEs occurred in 34.4% of subjects, at a rate per infusion of 0.029.

**Table.5:** Summary of adverse events occurring in the safety population (*N* = 61)

	*n*	%
Treatment-emergent adverse events	52	85.2
Suspected adverse drug reactions^a^	30	49.2
Adverse reactions^b^	19	31.1
Treatment-emergent serious adverse events^c^	7	11.5
Adverse events leading to withdrawal	4	6.6
Treatment-emergent adverse events during or within 72 h of an infusion	45	73.8
Infusion site reactions reported as a treatment-emergent adverse events	21	34.4
Serious bacterial infections^d^	1	1.6
Validated infections^d^	20	32.8
Any infection^d^	45	73.8

^a^Assessed as “definite,” “probable,” “possible,” or “doubtful/unlikely” by investigators

^b^Relationship assessed as “definite” by investigators

^c^None considered to be related to IGSC 20%

^d^Analyzed as efficacy endpoints and considered AEs

The most common TEAEs (≥ 10% incidence) during the IGSC 20% treatment phase were nasopharyngitis, infusion site erythema, infusion site pruritus, upper respiratory tract infection, cough, bronchitis, pyrexia, rhinitis, sinusitis, and headache (Table 6). Most TEAEs (97.1%) were mild or moderate in severity.

**Table.6:** TEAEs with ≥ 10% incidence and rate per infusion in the safety population

	Incidence (*N* = 61) *n* (%)	Rate per infusion^a^ (*N* = 3045 infusions)
TEAEs	52 (85.2)	0.135
Infusion site reactions	21 (34.4)	0.029
Nasopharyngitis	12 (19.7)	0.008
Infusion site erythema	10 (16.4)	0.009
Upper respiratory tract infection	9 (14.8)	0.006
Cough	9 (14.8)	0.003
Infusion site pruritus	8 (13.1)	0.006
Bronchitis	8 (13.1)	0.003
Headache	7 (11.5)	0.007
Rhinitis	7 (11.5)	0.003
Sinusitis	7 (11.5)	0.003
Pyrexia	7 (11.5)	0.002

*TEAE*, treatment-emergent adverse event

^a^Total number of events divided by the total number of infusions

## Discussion

This study was conducted to evaluate the efficacy, PK, and safety of IGSC 20% in subjects with PI. Its prospective, multi-center, open-label design was in accord with the EMA Guideline. On the primary efficacy endpoint, with only 1 subject experiencing an SBI during the study period, the incidence of SBIs was well below the prospectively defined efficacy criterion, and the efficacy of IGSC 20% was considered to have been demonstrated.

The findings from this study, the second with a caprylate/chromatography purified 20% SCIG product, are distinct from those of previous research [35, 36]; in the present study, there was no pharmacokinetic comparison to IV administration and no dose adjustment in the conversion of subjects from IV to SC administration. The observed IgG trough levels in the current study suggest that in this population—treatment-experienced subjects who were stably maintained on a prior IgG replacement regimen before entry—a DAF of 1:1 can provide comparable rates of SBIs, infections of any kind, and other infection parameters. This study also provides comprehensive data with regard to steady-state IgG levels, IgG subclasses, and specific pathogen antibody levels using a DAF of 1:1.

Regarding efficacy, our results are similar to those of a separate non-inferiority and safety study in subjects with PI [25], which was conducted to determine which weekly IGSC 20% dose produced steady-state *AUC* of total IgG that was non-inferior to that of IVIG (specifically Gamunex 10%) employing a DAF of 1.37 (vs 1.00) and treating subjects for 24 weeks (vs 52 weeks). For example, only 1 subject in each study had an SBI, for a per person per year rate of 0.017 in this study and 0.049 in the North American study (where the event was sepsis due to a cat bite [25]). Both studies also had 1 event of hospitalization due to infection and similar per person per year rates of hospitalization due to infection (0.017 vs 0.049) [25], which themselves were similar to per subject-year rates of hospitalization for infection reported in studies of Cuvitru (immune globulin subcutaneous [human], 20% solution [Ig20Gly]; Takeda Pharmaceutical Company Limited, Lexington, MA, USA) in subjects with PI (0.02– 0.04) (13,14, 34). In addition, the rate of all infections per person per year in both studies was nearly identical (2.397 vs 2.367) and comparable to rates reported for Cuvitru (2.41–4.38) [25, 26, 37] and Hizentra (immune globulin subcutaneous [human], 20% solution [Ig20Gly]; CSL Behring, LLC, King of Prussia, PA, USA) (2.76- 5.18) [20, 22, 38]. The broad similarity in results from these 2 IGSC 20% pivotal studies is particularly notable because IgG mean trough levels, which averaged 947.64 mg/dL, remained well above the protective threshold of 500 mg/dL, even though the mean weekly dose of IGSC 20% was lower with a DAF of 1 (125.5 mg/kg vs 178.9 mg/kg). Antibiotic usage in the current study was commensurate with rates reported for other licensed SC IgG products (Cuvitru, Hizentra, Gammagard, and Cutaquiq), as shown in Online Resource 1, and efficacy was comparable [17, 18, 23, 37, 38]. Additionally, treatment with IGSC 20% had no apparent effect on the proportions, levels, or concentrations of IgG subclasses, and it generated antibody levels that surpassed the protective thresholds for *H. Influenzae*, anti-*tetanus* toxoid, measles, and *S. pneumoniae* (noninvasive and invasive disease).

IGSC 20% was well tolerated during the year-long treatment phase over a broad range of infusion rates, and the overall TEAE profile was consistent with expectations of SCIG therapy in this subject population. No safety concerns in children, adolescents, and adults were identified, and no new safety signal was observed.

This study has strengths and limitations. Strengths include providing new information about an important treatment alternative for patients with primary immunodeficiency without a dose adjustment, strictly adhering to the validated analytic approach required by regulators, and enrolling a study population that was geographically diverse. The preponderance of white subjects and slight over-representation of males somewhat reduced the diversity of the population, but the cohort was recruited to be representative of the prevalence and distribution of the heritable disorders under study [39]. Including a broader distribution of infusion rates (ie, faster infusions) may also have improved understanding of the general applicability and practicality of more rapid infusions for patient convenience.

## Conclusions

At a mean dose 125.5 mg/kg/week with no dose adjustment (DAF 1:1), IGSC 20% treatment provided protection against SBIs and infections and demonstrated a good safety profile in adult and pediatric patients with PI. Protective antibody titers against *S. pneumoniae*, *H. influenzae*, *C. tetani* (tetanus), and measles were observed. The IGSC 20% product will provide an additional treatment option for patients with PI.

## Supplementary Information

Below is the link to the electronic supplementary material.

Supplementary file1 (DOCX 33 KB)

## Data Availability

The data and materials used in this study will be provided to qualified researchers on reasonable request.
