# Correction to: A Multi-center, Open-Label, Single-Arm Trial to Evaluate the Efficacy, Pharmacokinetics, and Safety and Tolerability of IGSC 20% in Subjects with Primary Immunodeficiency

**DOI:** 10.1007/s10875-022-01219-3

**Published:** 2022-02-16

**Authors:** Manuel Santamaria, Olaf Neth, Jo A. Douglass, Gergely Krivan, Robin Kobbe, Ewa Bernatowska, Sofia Grigoriadou, Claire Bethune, Anita Chandra, Gerd Horneff, Michael Borte, Anja Sonnenschein, Pavlina Kralickova, Silvia Sánchez Ramón, Daman D. Langguth, Luis Ignacio Gonzalez‑Granado, Laia Alsina, Montse Querolt, Rhonda Griffin, Carrie Hames, Elsa Mondou, Jeffrey Price, Ana Sanz, Jiang Lin

**Affiliations:** 1 Unidad de Inmunologia Clinica, Hospital Universitario, Reina Sofía, Facultad de Medicina, Universidad de Cordoba, Cordoba, Spain; 2 Pediatric Infectious Diseases, Rheumatology and Immunology Unit, Hospital Universitario Virgen del Rocío, Instituto de Biomedicina de Sevilla, Universidad de Sevilla/CSIC, Red de Investigación Traslacional en Infectología Pediátrica RITIP, IBiSSeville, Spain; 3 Department of Immunology and Allergy, The Royal Melbourne Hospital, Melbourne, VIC Australia; 4 Department of Medicine, The University of Melbourne, Melbourne, VIC Australia; 5 Paediatric Haematology and Stem Cell Transplantation, Department, Szent Laszlo Hospital, Budapest, Hungary; 6 First Department of Medicine, Division of Infectious Diseases, University Medical Center Hamburg-Eppendorf, Hamburg, Germany; 7 Department of Immunology, Children’s Memorial Health Institute, Warsaw, Poland; 8 Department of Immunology, The Royal London Hospital, Barts Health NHS Trust, London, UK; 9 Peninsula Immunology and Allergy Service, University Hospitals Plymouth, Plymouth, UK; 10 Department of Medicine, University of Cambridge, Cambridge, UK; 11 Asklepios Kinderklinik Sankt Augustin, Sankt Augustin, Germany; 12 University Hospital of Cologne, Cologne, Germany; 13 Klinikum St Georg GmbH, Klinik für Kinder‐und Jugendmedizin, Leipzig, Germany; 14 Department of Pediatric Immunology and Rheumatology, University Medical Center of Johannes Gutenberg University Mainz, Mainz, Germany; 15 Department of Allergology and Clinical Immunology, Faculty of Medicine, Charles University and University Hospital in Hradec Kralove, Hradec Kralove, Czechia; 16 Servicio de Inmunología, Hospital Clínico San Carlos, Madrid, Spain; 17 Department of Immunology, Sullivan Nicolaides Pathology, Brisbane, QLD Australia; 18 Primary Immunodeficiencies Unit, Hospital Universitario 12 de Octubre and Department of Public and Maternal‑Child Health, Faculty of Medicine, Complutense University of Madrid, Madrid, Spain; 19 Clinical Immunology and Primary Immunodeficiencies Unit, Pediatric Allergy and Clinical Immunology Department, Hospital Sant Joan de Déu, Planta 3 Consultes Externes, Passeig Sant Joan de Déu 2, Esplugues de Llobregat, 08950 Barcelona, Spain; 20 Institut de Recerca Sant Joan de Déu, Barcelona, Spain; 21 Universitat de Barcelona, Barcelona, Spain; 22 Grifols Bioscience Industrial Group, Sant Cugat del Vallès, Barcelona, Spain; 23 Grifols Bioscience R&D, Research Triangle Park, NC USA

**Correction to** **:** **Journal of Clinical Immunology**

https://doi.org/10.1007/s10875-021-01181-6

Due to typesetting mistake, the author’s corrections were missed.

The original version has been corrected.

