# Supplementary material for: A Multi‑Center, Open‑Label, Single‑Arm Trial to Evaluate the Efficacy, Pharmacokinetics, and Safety and Tolerability of IGSC 20% in Subjects with Primary Immunodeficiency
Source: J Clin Immunol. 2022 Jan 1;42(3):500–11. doi: 10.1007/s10875-021-01181-6 (PMC9016006; doi:10.1007/s10875-021-01181-6)
Supplement: Supplementary file 1 — Supplementary file1 (DOCX 33 KB) [file 10875_2021_1181_MOESM1_ESM.docx]

**A Multi-Center, Open-Label, Single-Arm Trial to Evaluate Efficacy, Pharmacokinetics, and Safety and Tolerability of IGSC 20% in Subjects with Primary Immunodeficiency**

Manuel Santamaria^1^, Olaf Neth^2^, Jo A Douglass^3^, Gergely Krivan^4^, Robin Kobbe^5^, Ewa Bernatowska^6^, Sofia Grigoriadou^7^, Claire Bethune^8^, Anita Chandra^9^, Gerd Horneff^10^, Michael Borte^11^, Anja Sonnenschein^12^, Pavlina Kralickova^13^, Silvia Sánchez Ramón ^14^, Daman Langguth D^15^, Luis Ignacio Gonzalez-Granado^16^, Laia Alsina^17^, Montse Querolt^18^, Rhonda Griffin^19^, Carrie Hames^19^, Elsa Mondou^19^, Jeffrey Price^19^, Ana Sanz^18^, Jiang Lin^19^

1. Unidad de Inmunologia Clinica, Hospital Universitario Reina Sofía, Facultad de Medicina, Universidad de Cordoba, Spain
2. Pediatric Infectious Diseases, Rheumatology and Immunology Unit, Hospital Universitario Virgen del Rocío, Instituto de Biomedicina de Sevilla, IBiS/ Universidad de Sevilla/CSIC, Red de Investigación Traslacional en Infectología Pediátrica RITIP, Seville, Spain
3. Department of Immunology and Allergy, The Royal Melbourne Hospital and the Department of Medicine, The University of Melbourne, Victoria, Australia
4. Paediatric Haematology and Stem Cell Transplantation Department, Szent Laszlo Hospital, Budapest, Hungary
5. First Department of Medicine, Division of Infectious Diseases, University Medical Center Hamburg-Eppendorf, Germany
6. Department of Immunology, Children’s Memorial Health Institute, Warsaw, Poland
7. Department of Immunology, The Royal London Hospital, Barts Health NHS Trust, London, United Kingdom
8. Peninsula Immunology and Allergy Service, University Hospitals Plymouth, Plymouth, United Kingdom
9. Department of Medicine, University of Cambridge, United Kingdom
10. Asklepios Kinderklinik Sankt Augustin, Sankt Augustin, University Hospital of Cologne, Cologne, Germany
11. Klinikum St Georg GmbH, Klinik für Kinder‐ und Jugendmedizin, Leipzig, Germany
12. Department of Pediatric Immunology and Rheumatology, University Medical Center of Johannes Gutenberg University Mainz, Mainz, Germany
13. Department of Allergology and Clinical Immunology, Faculty of Medicine, Charles University and University Hospital in Hradec Kralove, Hradec Kralove, Czechia
14. Servicio de Inmunología, Hospital Clínico San Carlos, Madrid, Spain
15. Department of Immunology, Sullivan Nicolaides Pathology, Queensland, Australia
16. Primary Immunodeficiencies Unit, Hospital Universitario 12 de Octubre and Department of Public and Maternal - Child Health, Faculty of Medicine, Complutense University of Madrid, Madrid, Spain
17. Clinical Immunology and Primary Immunodeficiencies Unit, Pediatric Allergy and Clinical Immunology Department, Hospital Sant Joan de Déu, Barcelona, Spain; Institut de Recerca Sant Joan de Déu, Barcelona, Spain; Universitat de Barcelona, Spain.
18. Grifols Bioscience Industrial Group, Sant Cugat del Vallès, Barcelona, Spain
19. Grifols Bioscience Research Group, Research Triangle Park, NC, United States

Antibiotic usage in this study (GTI1503) was within the range reported for other subcutaneous immune globulin products approved for primary immunodeficiency syndromes in Europe and North America, as shown in the table below.

**Days of Antibiotic Use Per Subject-Year for Subcutaneous Immune Globulins**^a^

| Product | Total Days Per Subject-Year |
| --- | --- |
| IGSC 20% (GTI1503) | 53.3^b^ (44.4 prophylactic + 8.9 treatment) |
| IGSC 20% (GTI1502) | 56.6^b^ (27.7 prophylactic + 28.9 treatment) |
| Gammagard Liquid 10% [1] | 50.2 |
| Hizentra (20%) [2, 3] | North American study: 48.5  European study: 72.75 |
| Cuvitru (20%) [4, 5] | North American study: 57.59  European study: 18.11 |
| Cutaquig (16.5%) [6] | 39.6 |
| IGSC 20%, Immune Globulin Subcutaneous (Human), 20% Caprylate/Chromatography Purified  ^a^For commercial products, days of antibiotic use per subject-year were based on published data.  ^b^Because the days of antibiotic use per subject-year were additive in studies GTI1502 and GTI1503, the total antibiotic use per subject-year was obtained by summing use reported for prophylaxis and treatment. The rate of antibiotic use was calculated as the total number of days of use divided by the total duration of exposure in years across all subjects. | |

**References**

1. Wasserman RL, Melamed I, Kobrynski L, Strausbaugh SD, Stein MR, Sharkhawy M et al. Efficacy, safety, and pharmacokinetics of a 10% liquid immune globulin preparation (GAMMAGARD LIQUID, 10%) administered subcutaneously in subjects with primary immunodeficiency disease. J Clin Immunol. 2011;31(3):323-31. doi:10.1007/s10875-011-9512-z.

2. Jolles S, Bernatowska E, de Gracia J, Borte M, Cristea V, Peter HH et al. Efficacy and safety of Hizentra((R)) in patients with primary immunodeficiency after a dose-equivalent switch from intravenous or subcutaneous replacement therapy. Clin Immunol. 2011;141(1):90-102. doi:10.1016/j.clim.2011.06.002.

3. Hagan JB, Fasano MB, Spector S, Wasserman RL, Melamed I, Rojavin MA et al. Efficacy and safety of a new 20% immunoglobulin preparation for subcutaneous administration, IgPro20, in patients with primary immunodeficiency. J Clin Immunol. 2010;30(5):734-45. doi:10.1007/s10875-010-9423-4.

4. Suez D, Stein M, Gupta S, Hussain I, Melamed I, Paris K et al. Efficacy, Safety, and Pharmacokinetics of a Novel Human Immune Globulin Subcutaneous, 20 % in Patients with Primary Immunodeficiency Diseases in North America. J Clin Immunol. 2016;36(7):700-12. doi:10.1007/s10875-016-0327-9.

5. Borte M, Kriván G, Derfalvi B, Maródi L, Harrer T, Jolles S et al. Efficacy, safety, tolerability and pharmacokinetics of a novel human immune globulin subcutaneous, 20%: a Phase 2/3 study in Europe in patients with primary immunodeficiencies. Clin Exp Immunol. 2017;187(1):146-59. doi:10.1111/cei.12866.

6. Kobayashi RH, Gupta S, Melamed I, Mandujano JF, Kobayashi AL, Ritchie B et al. Clinical efficacy, safety and tolerability of a new subcutaneous immunoglobulin 16.5% (Octanorm [Cutaquig®]) in the treatment of patients with primary immunodeficiencies. Front Immunol. 2019;10:40. doi:10.3389/fimmu.2019.00040.
